# The Reaction Thermodynamics during Plating Al on Graphene Process

**DOI:** 10.3390/ma12020330

**Published:** 2019-01-21

**Authors:** Zhanyong Zhao, Peikang Bai, Liang Li, Jing Li, Liyun Wu, Pengcheng Huo, Le Tan

**Affiliations:** School of Materials Science and Engineering, North University of China, Taiyuan 030051, China; syuzzy@126.com (Z.Z.); nucliliang@126.com (L.L.); jing.li3d@hotmail.com (J.L.); wuliyunnuc@126.com (L.W.); nuchpc@126.com (P.H.); hltanle@foxmail.com (L.T.)

**Keywords:** density functional theory (DFT), thermodynamics, graphene/aluminum composites

## Abstract

This research explored a novel chemical reduction of organic aluminum for plating Al on a graphene surface. The thermodynamics of the Al plating reaction process were studied. The Al plating process consisted of two stages: the first was to prepare (C_2_H_5_)_3_Al. In this reaction, the ΔH(enthalpy) was 10.64 kcal/mol, the ΔG(Gibbs free energy) was 19.87 kcal/mol and the ΔS(entropy) was 30.9 cal/(mol·K); this was an endothermic reaction. In the second stage, the (C_2_H_5_)_3_Al decomposed into Al atoms, which were gradually deposited on the surface of the graphene and the Al plating formed. At 298.15 K, the ΔH was −20.21 kcal/mol, the ΔG was −54.822 kcal/mol, the ΔS was 116.08 cal/(mol·K) and the enthalpy change was negative, thus indicating an endothermic reaction.

## 1. Introduction

Graphene/aluminum composites have high strength, high conductivity and high toughness. Thus, graphene/aluminum composites have wide application potentiality in the electronics, automotive and aerospace industry [1,2,3,4,5,6,7,8]. However, graphene/aluminum composites are difficult to prepare; because of the poor wettability between Al and graphene, the graphene aggregates easily in the Al matrix, which can decrease the mechanical properties of the composites [9,10]. In order to improve the wettability between graphene and Al, the ideal method is to coating melt on the surface of the graphene, by methods including self-assembly, chemical reduction, electrochemical deposition, redox method and chemical vapor deposition. For instance, Bagheri et al. prepared graphene-gold nanocomposites by self-assembly [11]. Tsai et al. coated the Cu nanoparticles on the graphene surface through coalescence and epitaxial self-assembly and studied molecular dynamics during the process [12]. Muszynski et al. synthesized gold nanoparticles through the chemical reduction of AuCl_4_^−^ (Aldrich) with NaBH_4_ and coated the gold nanoparticles on the surface of graphene [13]. Zhao et al. prepared graphene nanoplatelets by reinforcing copper matrix composites with electrochemical deposition and the composites’ hardness and conductivity reached 111.2 HV and 89.2% IACS [14]. Kim et al. prepared single-atomic-layer graphene film on the surface of Cu through chemical vapor deposition (CVD), then obtained multi-layer graphene/copper composites with the strength of 1.5 GPa [15]. According to previous investigations, gold, copper, or nickel nanoparticles were usually coated on the graphene. However, these metal nanoparticles may be viewed as impurities in Al alloys, which can affect their properties. Plating Al on graphene is an effective method to improve graphene’s wettability and reduce these impurities. Because the Al is active, it is difficult to displace Al atom from conventional Al salt solution [16]. 

Selective laser melting (SLM), through melting successive layers of metal powder, is a promising metal additive manufacturing method [17,18], it has huge advantages compared to traditional processing methods [19,20,21,22,23,24] and therefore has been widely used in the fields of medical, military, aerospace and automobile manufacturing [25]. The purpose of this new method is to increase the weight of graphene by plating Al on its surface, which solves the problem of uneven dispersion of graphene in Al powder for SLM. Considering that plating Al on graphene is difficult, we explored a novel chemical reduction of organic aluminum for plating Al on the graphene surface [19]. The Al plating process consisted of two stages. In the first stage, the Al powders were added to the C_2_H_5_Br solution, to produce (C_2_H_5_)_3_Al. In the second stage, the (C_2_H_5_)_3_Al decomposed into Al atoms, which were gradually deposited on the surface of the graphene and the Al plating formed [26]. The microstructure evolution was reported [26]. 

However, the reaction mechanism was unclear, especially the thermodynamics of the reaction process. Density functional theory (DFT) is a quantum mechanical method for studying the electronic structure of multi-electron systems [27,28,29]. DFT has a wide range of applications in physics and chemistry, especially for studying the properties of molecules and condensed states [30,31,32]. It is one of the most commonly used methods in computational materials and computational chemistry [30]. The objective of the study described here is to elucidate the thermodynamics of the Al plating reaction process and it provides guidance for process optimization.

## 2. Experiment and Simulation 

### 2.1. Experiment

**Figure 1:**
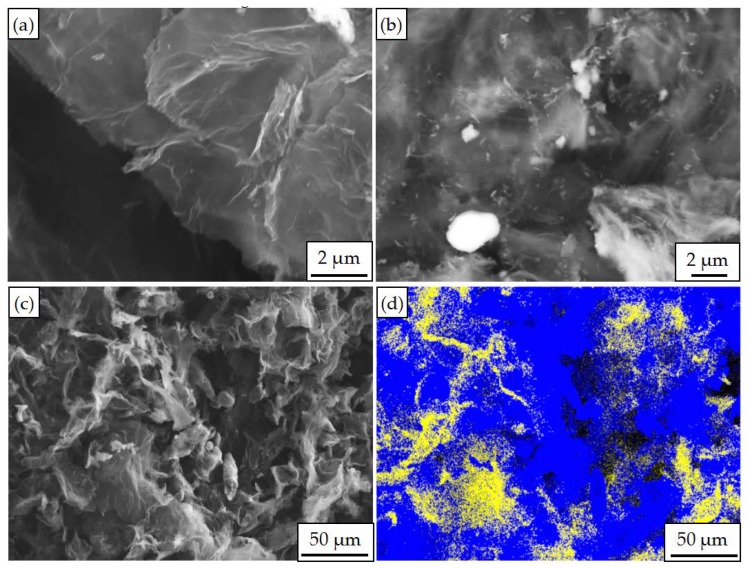
Scanning electron microscopy (SEM) image and energy dispersive spectrometer (EDS) analysis of Graphene/aluminum composites with different reaction times: (**a**) Graphene/aluminum composites prepared with reaction time of 1 h; (**b**) Graphene/aluminum composites prepared with reaction time of 1.5 h; (**c**) Low-magnification SEM image of composite prepared with reaction time of 1.5 h; (**d**) map analysis of (**c**).

The Al powders and the graphene were employed as raw materials, as shown in Figure 1. During the Al plating reaction process, H_2_ gas was pumped into the reaction vessel and the Al powder (1.5 g), aluminum chloride (0.1 g) and iodine (0.1 g) were dried and added into the C_2_H_5_Br (29 mL) at 39 °C. Al reacted with C_2_H_5_Br and the (C_2_H_5_)_2_AlBr and C_2_H_5_AlBr_2_ were obtained as follows [26]:

(1) $$3C_{2}H_{5}\mathrm{Br}+2\mathrm{Al}\to {\left(C_{2}H_{5}\right)}_{2}\mathrm{AlBr}+C_{2}H_{5}\mathrm{AlBr}$$

The Al reacted with C_2_H_5_AlBr_2_ to produce (C_2_H_5_)_2_AlBr, Al and AlBr_3_ [26]:

(2) $$2C_{2}H_{5}\mathrm{AlBr}+\mathrm{Al}\to {\left(C_{2}H_{5}\right)}_{2}\mathrm{AlBr}+\mathrm{Al}+{\mathrm{AlBr}}_{3}$$

The (C_2_H_5_)_2_AlBr and Al further reacted to produce Al, (C_2_H_5_)_3_Al and AlBr_3_ via Equation [26]:

(3) $$3{\left(C_{2}H_{5}\right)}_{2}\mathrm{AlBr}+\mathrm{Al}\to 2{\left(C_{2}H_{5}\right)}_{3}\mathrm{Al}+\mathrm{Al}+{\mathrm{AlBr}}_{3}$$

After reaction, the solution temperature was kept at 0 °C for 1 h. The tetrahydrofuran was added to the solution. The solution was filtered after the reaction and the alkyl aluminum solution was obtained. Then, the graphene (0.05 g) was added to the alkyl aluminum solution. The temperature was kept at 70–100 °C for 1–1.5 h and the (C_2_H_5_)_3_Al was decomposed into Al, H_2_ and C_2_H_4_ [26]:

(4) $$2{\left(C_{2}H_{5}\right)}_{3}\mathrm{Al}\to 6C_{2}H_{4}+3H_{2}+2\mathrm{Al}$$

The Al atoms were gradually deposited on the surface of the graphene and the Al plating formed. Al atoms absorbed on graphene may form upon (C_2_H_5_)_3_Al/graphene collisions. This reaction is initiated by ethane elimination from the (C_2_H_5_)_3_Al molecule, similar to the observations reported for (CH_3_)_3_Al/graphene [33].

Microstructure observation was carried out using a scanning electron microscope (SEM) (Zeiss Ultra 55, Carl Zeiss Microscopy, Jena, Germany) equipped with energy dispersive spectroscopy (EDS).

### 2.2. Computation Details

During the process of plating Al on the graphene, the thermodynamics of the chemical reduction of organic aluminum were simulated by density functional theory (DFT) methods implemented in the DMol3 package of Materials Studio. The structure of the reaction products was analyzed through the DFT, revealing the thermodynamic properties and reaction types of the chemical reactions. Spin-unrestricted DFT in the generalized gradient approximation (GGA) with the Revised Perdew-Burke-Eruzerhof (RPBE) exchange-correlation functional approach and double numerical plus polarization atomic orbitals was employed as the basis set. The Brillouin zone was sampled using the 4 × 4 × 1 k-point grid thickness, which presented a good approximation of the model below the article. In addition, the energy tolerance accuracy, maximum force and displacement were set as 1 × 10^−5^ Ha, 2 × 10^−3^ Ha/Å and 5 × 10^−3^ Å, respectively, to ensure high accuracy in all calculations.

During the Dmol3 simulation process, the relationship between thermodynamic properties (entropy S, enthalpy H, heat capacity Cp, Gibbs free energy G) and temperature can be calculated from the vibration frequency. The total energy at 0 K was obtained during the simulation. The translational energy, rotational energy and vibration energy were used to calculate the thermodynamic properties at an instantaneous temperature. The instantaneous enthalpy H is:

(5) $$H\left(T\right)=E_{vib}\left(T\right)+E_{rot}\left(T\right)+E_{trans}\left(T\right)+RT$$

where $E_{vib}\left(T\right)$, $E_{rot}\left(T\right)$ and $E_{trans}\left(T\right)$ are vibration energy, rotational energy and translational energy respectively at temperature *T* and *R* is an ideal gas constant.

The contribution of vibration to enthalpy is:

(6) $$E_{vib}\left(T\right)=\frac{R}{K}\frac{1}{2}\underset{i}{\sum }hv_{i}+\frac{R}{K}\underset{i}{\sum }\frac{hv_{i}\exp\left(-hv_{i}/kT\right)}{1-\exp\left(-hv_{i}/kT\right)}$$

The contribution of vibration to entropy is:

(7) $$S_{vib}=R\underset{i}{\sum }\frac{\left(hv_{i}/kT\right)\exp\left(-hv_{i}/kT\right)}{1-\exp\left(-hv_{i}/kT\right)}-R\underset{i}{\sum }\ln\left[1-\exp\left(-hv_{i}/kT\right)\right]$$

The contribution of vibration to heat capacity at normal pressure is:

(8) $$C_{vib}=R\underset{i}{\sum }\frac{{\left(hv_{i}/kT\right)}^{2}\exp\left(-hv_{i}/kT\right)}{{\left[1-\exp\left(-hv_{i}/kT\right)\right]}^{2}}$$

where *k* is the Boltzmann constant, *h* is the Planck constant and *v_i_* is the vibration frequency of the *i*th atom. Each of the chemical bond vibrational frequencies was calculated by DFT at 298.15 K and then assumed to remain constant with Temperature.

## 3. Results and Discussions

### 3.1. Preparation and Reaction Mechanism of Al-Coated Graphene

During the Al plating process, with the increase of reaction time, more Al was deposited on the graphene, as shown in Figure 1a,b. When the chemical reduction reaction was at 1.5 h, abundant Al atoms were deposited on the graphene uniformly, the Al plating was formed and the content of the Al element was 71%, as shown in Figure 1c,d.

### 3.2. Reaction Thermodynamics during Plating Al on Graphene Process

The molecular model of each substance was established and its structure optimized during the chemical reduction reaction process. The vibration frequency was calculated and the thermodynamic properties of each substance were analyzed. The thermodynamics of the formation and decomposition of (C_2_H_5_)_3_Al were calculated according to the laws of thermodynamics.

During the process of plating Al on grapheme, based on the reaction Equations (1) and (3), the structural optimization and thermodynamic calculation of C_2_H_5_Br, (C_2_H_5_)_2_AlBr, C_2_H_5_AlBr_2_, (C_2_H_5_)_3_Al and AlBr_3_ were carried out through the Al cluster (Al_3_) molecular model [34].

#### 3.2.1. Structure Optimization and Thermodynamic Properties of C_2_H_5_Br

**Figure 2:**
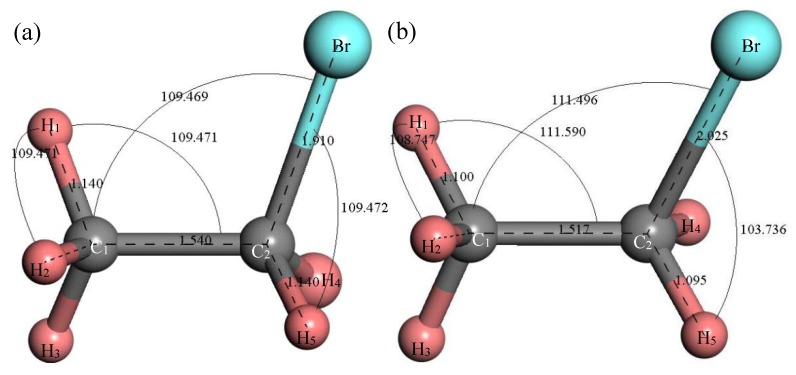
Molecular model of C_2_H_5_Br molecule. (**a**) Initial model; (**b**) Optimized model. The unit of the angle in the image is (°), and the unit of the bond length is angstrom (Å).

Figure 2 shows the structure of the C_2_H_5_Br molecule. The initial structure of the C_2_H_5_Br molecule built in MS is shown in Figure 2a; the stable molecular structure after structure optimization is shown in Figure 2b. After structure optimization, ∠H_1_C_1_C_2_ was reduced from 109.471° to 108.747°, ∠H_1_C_1_C_2_ increased from 109.471° to 111.590°, ∠C_1_C_2_Br increased from 109.469° to 111.496° and ∠BrC_2_H_5_ decreased from 109.472° to 103.736°. The bond length of H_1_–C_1_ decreased from 1.14 Å to 1.1 Å, the C_1_–C_2_ bond was reduced from 1.54 Å to 1.517 Å, the C_2_–Br bond as increased from 1.91 Å to 2.025 Å and the C_2_–H_5_ bond was reduced from 1.14 Å to 1.095 Å. During the structure optimization process, the bond angle and bond length of atoms tended to be stable through the vibration displacement and the total energy was gradually minimized.

**Figure 3:**
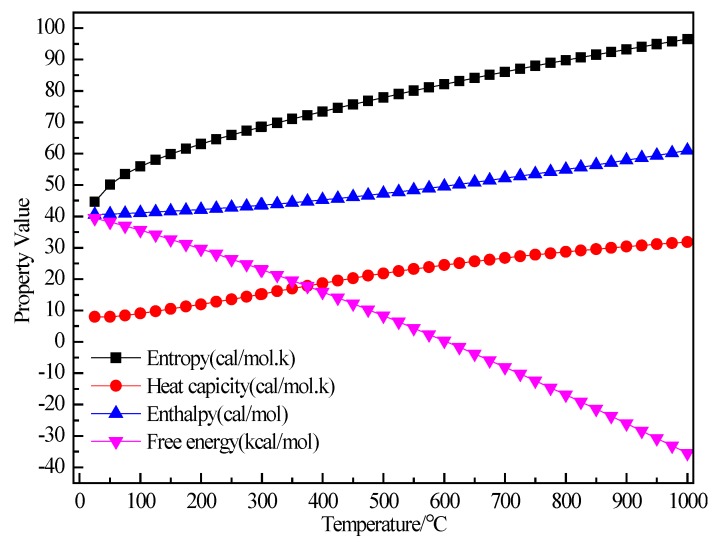
The relationship between the thermodynamic properties of C_2_H_5_Br and temperature.

Figure 3 shows the relationship between the thermodynamic properties (entropy S, enthalpy H, heat capacity Cp, Gibbs free energy G) of the C_2_H_5_Br and temperature was obtained through Equations (5)–(8). In the range of 25–1000 K, the enthalpy of the C_2_H_5_Br molecule had a linear relationship with the temperature and the enthalpy value increased with the increase of temperature. The heat capacity of C_2_H_5_Br gradually increased with the increase of temperature, although the free energy decreased. At 298.15 K, the enthalpy, entropy, free energy and heat capacity of C_2_H_5_Br molecules were 43.533 kcal/mol, 68.433 cal/(mol·K), 15.174 cal/(mol·K) and 23.127 kcal/mol respectively, as shown in Table 1.

#### 3.2.2. Structure Optimization and Thermodynamic Properties of (C_2_H_5_)_2_AlBr

**Figure 4:**
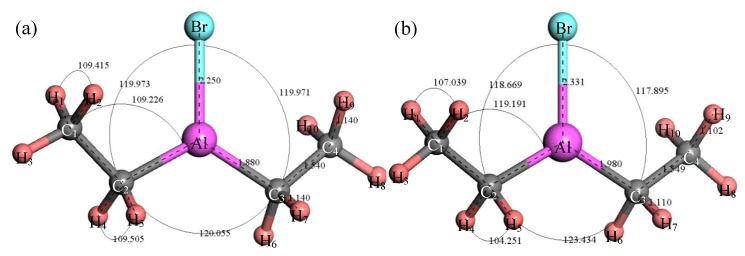
Molecular model of (C_2_H_5_)_2_AlBr molecule. (**a**) Initial model; (**b**) Optimized model. The unit of the angle in the image is (°), and the unit of the bond length is angstrom (Å).

**Figure 5:**
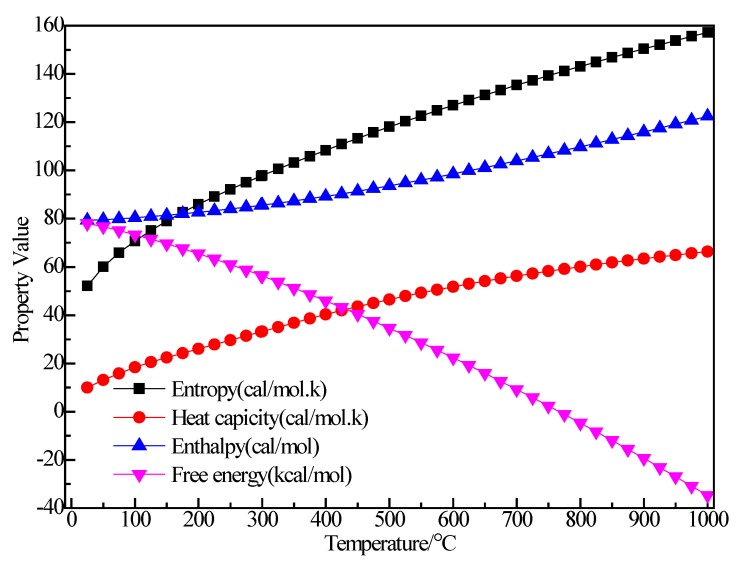
The relationship between the thermodynamic properties of (C_2_H_5_)_2_AlBr and temperature.

Figure 4 shows the structure of (C_2_H_5_)_2_AlBr. After structure optimization, ∠H_1_C_1_H_2_ decreased from 109.415° to 107.039°, ∠C_1_C_2_Al increased by 9.965°, ∠H_4_C_2_H_5_ decreased by 5.254° and ∠C_2_AlC_3_ increased by 2.571°. The length of the Br–Al bond increased from 2.250 Å to 2.331 Å, the length of the Al–C_3_ bond increased from 1.88 Å to 1.98 Å, the length of the C_3_–H_7_ bond decreased by 0.03 Å, the length of the C_3_–C_4_ bond increased by 0.009 Å and the length of the C_4_–H_9_ bond decreased from 1.140 Å to 1.102 Å. Figure 5 shows the relationship between the thermodynamic properties of (C_2_H_5_)_2_AlBr and temperature. It can be seen that the enthalpy, entropy and heat capacity of (C_2_H_5_)_2_AlBr increased with the increase of temperature in the range of 25–1000 K and the free energy decreased with the increase of temperature. At 298.15 K, the enthalpy, entropy, heat capacity and free energy were 85.548 kcal/mol, 97.648 cal/(mol·K), 33.078 cal/(mol·K) and 56.435 kcal/mol, respectively, as shown in Table 1. 

#### 3.2.3. Structure Optimization and Thermodynamic Properties of C_2_H_5_AlBr_2_

**Figure 6:**
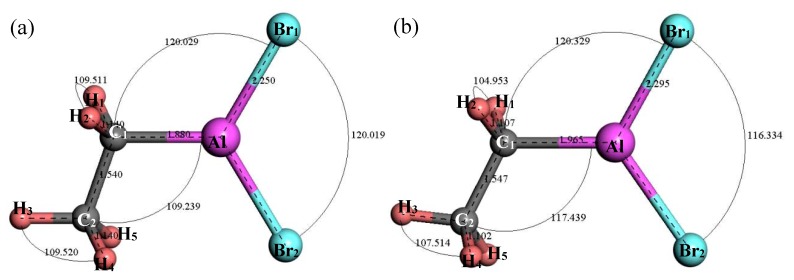
Molecular model of C_2_H_5_AlBr_2_ molecule. (**a**) Initial model; (**b**) Optimized model. The unit of the angle in the image is (°), and the unit of the bond length is angstrom (Å).

**Figure 7:**
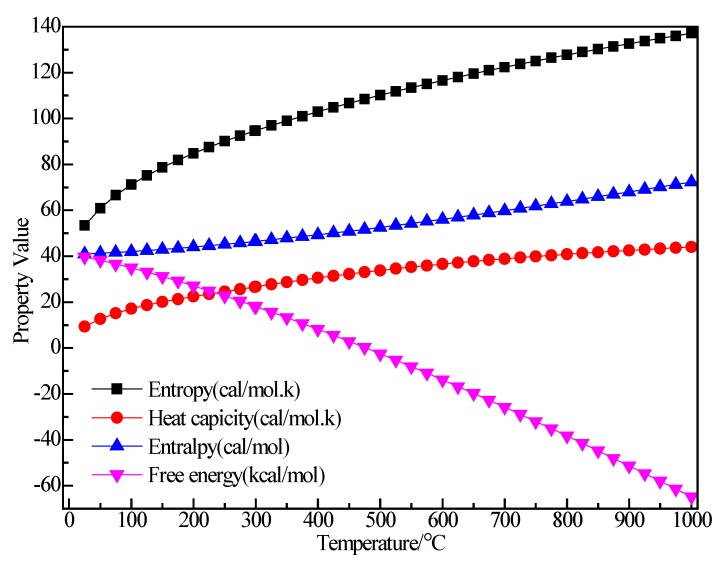
The relationship between the thermodynamic properties of C_2_H_5_AlBr_2_ and temperature.

Figure 6 shows the original and the optimal structure of C_2_H_5_AlBr_2_. After structure optimization, ∠H_1_C_1_H_2_ decreased from 109.511° to 104.953°, ∠H_3_C_2_H_4_ decreased from 109.52° to 107.514°, ∠C_2_C_1_Al increased from 109.239° to 117.439°, ∠C_1_AlBr_1_ increased by 0.3° and ∠Br_2_AlBr_1_ decreased by 3.685°. The bond length of H_2_–C_1_ decreased from 1.14 Å to 1.102 Å and the bond length of C_1_–C_2_ increased by 0.007 Å, indicating that the C–C bond was relatively stable. The bond length of C_1_–Al was increased by 0.085 Å and the bond length of Al-Br_1_ was increased by 0.45 Å. Figure 7 shows the relationship between the thermodynamic properties of C_2_H_5_AlBr_2_ and temperature. It can be seen that the enthalpy, entropy and heat capacity of C_2_H_5_AlBr_2_ increased with the increase of temperature in the range of 25–1000 K. The free energy decreased with the increase of temperature. At 298.15 K, the enthalpy, entropy, heat capacity and free energy were 46.425 kcal/mol, 94.579 cal/(mol·K), 26.606 cal/(mol·K) and 18.226 kcal/mol respectively (Table 1).

#### 3.2.4. Structure Optimization and Thermodynamic Properties of (C_2_H_5_)_3_Al

**Figure 8:**
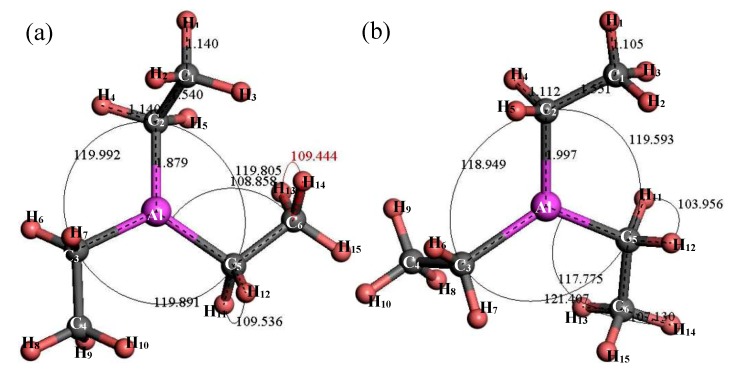
Molecular model of (C_2_H_5_)_3_Al molecule. (**a**) Initial model; (**b**) Optimized model. The unit of the angle in the image is (°), and the unit of the bond length is angstrom (Å).

**Figure 9:**
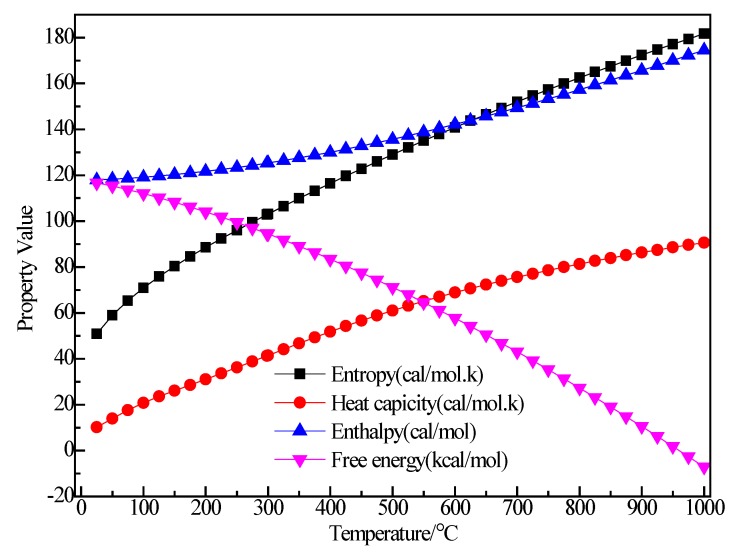
The relationship between the thermodynamic properties of (C_2_H_5_)_3_Al and temperature.

Figure 8 shows the structure of (C_2_H_5_)_3_Al. After structure optimization, ∠C_2_AlC_3_ was reduced from 119.992° to 118.949°, ∠C_2_AlC_5_ decreased from 119.805° to 119.593°, ∠C_3_AlC_5_ increased from 119.891° to 121.407°, ∠AlC_5_C_6_ increased from 108.858° to 117.775°, ∠H_11_C_5_H_12_ decreased from 109.536° to 103.956° and ∠H_13_C_6_H_14_ decreased from 109.444° to 107.13°. The H_1_–C_1_ bond length was reduced from 1.14 Å to 1.105 Å, the C_1_–C_2_ bond length increased from 1.54 Å to 1.551 Å, the C_2_–Al bond length increased from 1.879 Å to 1.997 Å and the C_2_–H_4_ bond length was reduced from 1.14 Å to 1.112 Å. During the optimization process, the C–Al bond rotated, the bond angle had large variation, the bond length changed little and the initial structure was significantly different from the optimized structure. Figure 9 shows the thermodynamic properties of (C_2_H_5_)_3_Al. In the range of 25–1000 K, the enthalpy, entropy and heat capacity of (C_2_H_5_)_3_Al increased with the increase of temperature. The free energy decreased with the increase of temperature. At 298.15 K, the enthalpy, entropy and heat capacity were 125.294 kcal/mol, 102.836 cal/(mol·K), 41.264 cal/(mol·K) and 94.634 kcal/mol, respectively.

#### 3.2.5. Structure Optimization and Thermodynamic Properties of AlBr_3_

**Figure 10:**
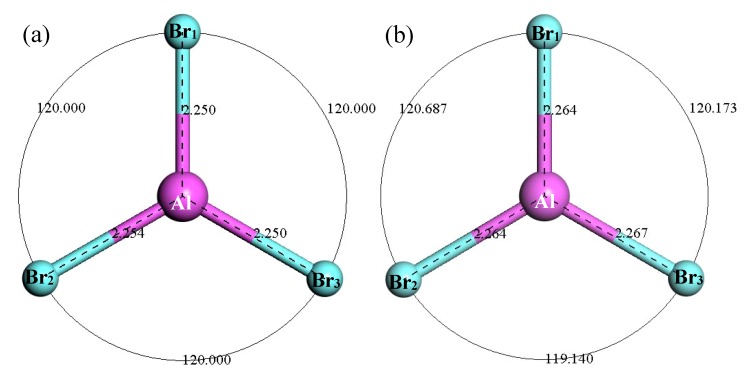
Molecular model of AlBr_3_ molecule. (**a**) Initial model; (**b**) Optimized model. The unit of the angle in the image is (°), and the unit of the bond length is angstrom (Å).

**Figure 11:**
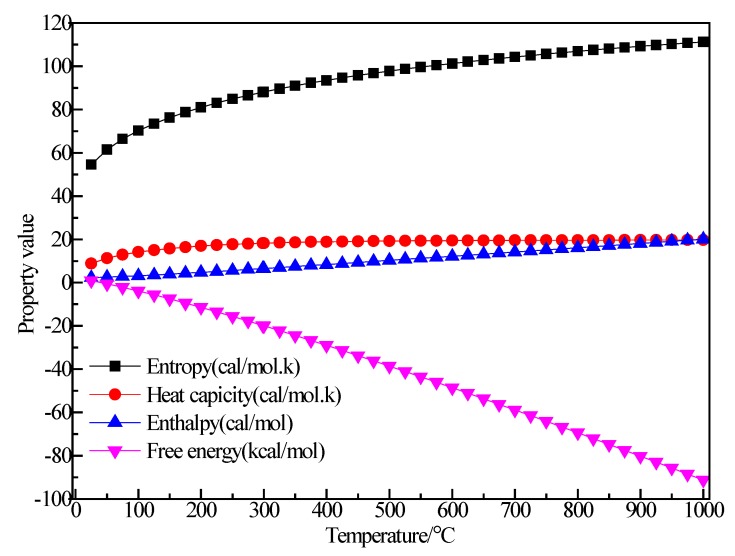
The relationship between the thermodynamic properties of AlBr_3_ and temperature.

Figure 10 shows the structure of AlBr_3_. It can be seen that after optimization of the AlBr_3_ structure, the bond angle of AlBr_3_ increased from equal 120° to 120.687°, 120.173° and 119.14°. The Br_1_–Al bond length increased from 2.25 Å to 2.264 Å, the Br_2_–Al bond length increased from 2.254 Å to 2.264 Å and the Br_3_–Al bond length increased from 2.25 Å to 2.267 Å. Figure 11 shows the thermodynamic properties of AlBr_3_. It can be seen that in the range of 25–1000 K, the enthalpy and entropy of the AlBr_3_ molecule increased with the increase of temperature, the heat capacity tended to be stable with the increase of temperature and the free energy decreased with the increase of temperature. The free energy was 0 kcal/mol at 25 K, which gradually decreased to a negative value with the increase of temperature. At 298.15 K, the enthalpy, entropy, heat capacity and free energy were 6.478 kcal/mol, 88.04 cal/(mol·K), 18.250 cal/(mol·K) and −19.771 kcal/mol, respectively.

Table 2 shows the thermodynamic properties during Al reacting with C_2_H_5_Br to produce (C_2_H_5_)_2_AlBr and C_2_H_5_AlBr_2_. It can be seen that when the reaction temperature was 298.15 K, the ΔH was −160.77 kcal/mol, ΔG was −139.83 kcal/mol and ΔS was −70.2 cal/(mol·K); it was thus an exothermic reaction.

Table 3 shows the total energy and thermodynamic properties of each component during (C_2_H_5_)_3_Al preparation at 298.15 K. Table 4 shows the thermodynamic properties during the (C_2_H_5_)_3_Al preparation process (reaction Equation (3)). At 298.15 K, the ΔH was 10.64 kcal/mol, the ΔG was 19.87 kcal/mol, the ΔS was 30.9 cal/(mol·K) and the enthalpy change was greater than 0; indicating this was an endothermic reaction.

#### 3.2.6. Structure Optimization and Thermodynamic Properties of C_2_H_4_

**Figure 12:**
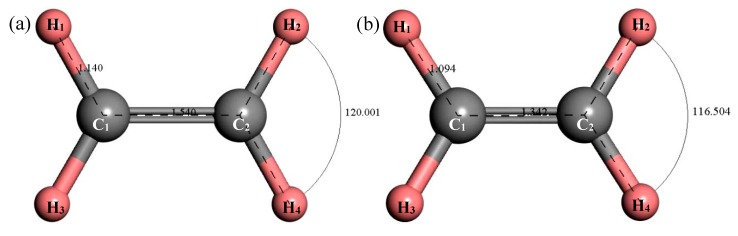
Molecular model of C_2_H_4_ molecule. (**a**) Initial model; (**b**) Optimized model. The unit of the angle in the image is (°), and the unit of the bond length is angstrom (Å).

**Figure 13:**
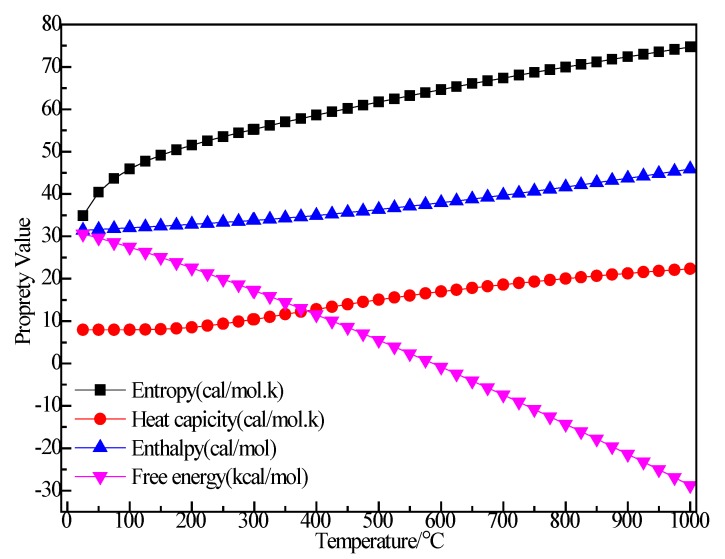
The relationship between the thermodynamic properties of C_2_H_4_ and temperature.

Figure 12 shows the structure of C_2_H_4_. After structural optimization, ∠H_2_C_2_H_4_ was reduced from 120.001° to 116.504°. The bond length of C–H decreased from 1.14 Å to 1.094 Å and the bond length of C=C was reduced from 1.54 Å to 1.342 Å. Figure 13 shows the thermodynamic properties of C_2_H_4_. It can be seen that the enthalpy, entropy and heat capacity of C_2_H_4_ increased with the increase of temperature in the range of 25–1000 K. The free energy decreased with the increase of temperature. At 298.15 K, the enthalpy, entropy, heat capacity and free energy were 33.759 kcal/mol, 55.228 cal/(mol·K), 10.372 cal/(mol·K) and 17.293 kcal/mol respectively (Table 5).

#### 3.2.7. Structure Optimization and Thermodynamic Properties of H_2_

**Figure 14:**
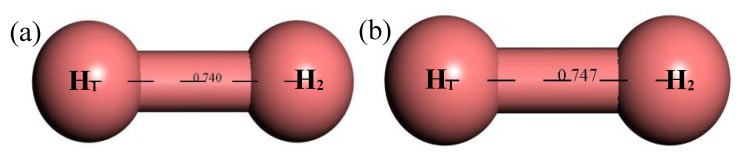
Molecular model of H_2_ molecule. (**a**) Initial model; (**b**) Optimized model. The unit of the angle in the image is (°), and the unit of the bond length is angstrom (Å).

**Figure 15:**
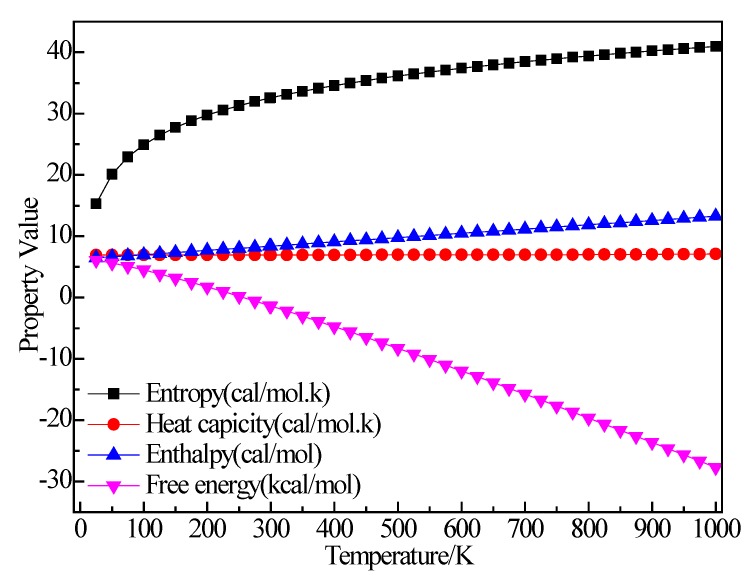
The relationship between the thermodynamic properties of H_2_ and temperature.

Figure 14 shows the structure of H_2_. It can be seen that after structural optimization, the bond length of H–H increased from 0.74 Å to 0.747 Å. Figure 15 shows the relationship between the thermodynamic properties of H_2_ and temperature. The enthalpy, entropy and heat capacity of H_2_ increased with the increase of temperature and the free energy decreased with the increase of temperature. At 298.15 k, the enthalpy, entropy, heat capacity and free energy were respectively 8.367 cal/mol, 32.531 cal/(mol·K), 6.955 cal/(mol·K) and −1.332 kcal/mol (Table 5).

Table 5 shows the thermodynamic properties during the (C_2_H_5_)_3_Al decomposition process (reaction Equation (4)). At 298.15 K, the ΔH was −20.21 kcal/mol, the ΔG was −54.822 kcal/mol, the ΔS was 116.08cal/(mol·K) (Table 6) and the enthalpy change was less than 0, this was an endothermic reaction.

## 4. Conclusions

We explored a novel chemical reduction of organic aluminum for plating Al on a graphene surface. The thermodynamics of the Al plating reaction process were studied. The Al plating process consisted of two stages: the first was to prepare (C_2_H_5_)_3_Al; the ΔH was 10.64 kcal/mol, the ΔG was 19.87 kcal/mol, the ΔS was 30.9 cal/(mol·K); this was an endothermic reaction. In the second stage, the (C_2_H_5_)_3_Al decomposed into Al atoms, which were gradually deposited on the surface of the graphene and the Al plating formed. At 298.15 K, the ΔH was −20.21 kcal/mol, the ΔG was −54.822 kcal/mol, the ΔS was 116.08 cal/(mol·K) and the enthalpy change was negative, thus indicating an endothermic reaction. The results show that the reaction efficiency can be improved significantly by increasing the reaction temperature and reaction time appropriately.

## Figures and Tables

**Table 1:** The total energy and the thermodynamic properties at 298.15 K of each component.

Substance	E (^1^Har/at)	H (kcal/mol)	G (kcal/mol)
C_2_H_5_Br	−423.7794203	43.533	23.127
Al	−727.1831592	4.348	−16.613
(C_2_H_5_)_2_AlBr	−745.5311183	85.548	56.435
C_2_H_5_AlBr_2_	−1010.8496025	46.425	18.226

^1^ 1 Har/at = 627.5 kcal/mol.

**Table 2:** The thermodynamic properties during Al reacting with C_2_H_5_Br to produce (C_2_H_5_)_2_AlBr and C_2_H_5_AlBr_2._

^1^ΔH (kcal/mol)	^2^ΔG (kcal/mol)	^3^ΔS (cal/mol·K)
−160.77	−139.83	−70.2

^1^$\Delta H=\sum {\left(E+H\right)}_{product}-\sum {\left(E+H\right)}_{reactant}$; ^2^$\Delta G=\sum {\left(E+H\right)}_{product}-\sum {\left(E+H\right)}_{reactant}$; ^3^$\Delta S=\frac{\left(\Delta H-\Delta G\right)}{T}$.

**Table 3:** Total energy and thermodynamic properties of each component during (C_2_H_5_)_3_Al preparation at 298.15 K.

Substance	E (Har/at)	H (kcal/mol)	G (kcal/mol)
(C_2_H_5_)_3_Al	−480.2022142	125.294	94.634
AlBr_3_	−1276.1579299	6.478	−19.771

**Table 4:** The thermodynamic properties during preparing (C_2_H_5_)_3_Al process.

ΔH (kcal/mol)	ΔG (kcal/mol)	ΔS (cal/mol·K)
10.64	19.87	30.9

**Table 5:** The total energy and thermodynamic properties of each component during (C_2_H_5_)_3_Al decomposition at 298.15 K.

Substance	E (Har/at)	H (kcal/mol)	G (kcal/mol)
C_2_H_4_	−78.6243401	33.759	17.293
H_2_	−1.2899789	8.367	−1.332

**Table 6:** The thermodynamic properties during preparing (C_2_H_5_)_3_Al process.

ΔH (kcal/mol)	ΔG (kcal/mol)	ΔS (cal/mol·K)
−20.21	−54.822	116.08

## References

[1] Boostani A.F., Yazdani S., Khosroshahi R.A., Jiang Z.Y., Wei D. (2018). A novel graphene-stimulated semi-solid processing to fabricate advanced aluminium matrix nanocomposites. Mater. Sci. Eng. A.

[2] Hu Z., Chen F., Xu J.L., Nian Q., Lin D., Chen C.J., Zhu X., Chen Y., Zhang M. (2018). 3D printing graphene-aluminum nanocomposites. J. Alloys Compd..

[3] Wen Z.Q., Zhao Y.H., Li H.J., Zhang Y.M., Wang S., Hou H. (2018). Theoretical Calculations of the Ideal Strength of Ni, NiAl and Ni_3_Al in Tension and Shear. Sci. Adv. Mater..

[4] Jia J.G., Liu D.Q., Gao C.Q., Ji G.S., Guo T.M. (2018). Preparation and mechanical properties of short carbon fibers reinforced alpha-Al_2_O_3_-based composites. Ceram. Int..

[5] Wang X.W., Zhu X.J., Gao J.H., Zheng Z.Z., Wang H.J. (2018). Milling Research and Tool Selection Design of SiC_14_Cu_4_Mg_0.5_Si based on Aluminium Matrix 2A14. J. Wuhan Univ. Technol..

[6] Zhang J.J., Li H.T., Liu H., Wang X.J., Ma Y., Wang N., Umar A., Guo Z.H. (2018). Determining Interfacial Shear Bond Strength in Thin Laminated Metal Composites. Sci. Adv. Mater..

[7] Zhao Y.H., Deng S.J., Liu H., Zhang J.X., Guo Z.H., Hou H. (2018). First-principle investigation of pressure and temperature influence on structural, mechanical and thermodynamic properties of Ti(3)AC(2) (A = Al and Si). Comput. Mater. Sci..

[8] Chen G., Chen W., Zhang G.W., Zheng S.Q., Zhang Z.M. (2018). Microstructures and Mechanical Properties of Al-12Zn2.4Mg-1.2Cu Alloy under Different Deformation Ways. Rare Met. Mater. Eng..

[9] Gao X., Yue H.Y., Guo E.J., Zhang H., Lin X.Y., Yao L.H., Wang B. (2016). Preparation and tensile properties of homogeneously dispersed graphene reinforced aluminum matrix composites. Mater. Des..

[10] Zhao M., Xiong D.B., Tan Z.Q., Fan G.L., Guo Q., Guo C.P., Li Z.Q., Zhang D. (2017). Lateral size effect of graphene on mechanical properties of aluminum matrix nanolaminated composites. Scr. Mater..

[11] Bagheri P., Farivar M., Simchi A. (2018). Graphene-mediated self-assembly of gold nanorods into long fibers with controllable optical properties. Mater. Lett..

[12] Tsai P.C., Jeng Y.R. (2019). Coalescence and epitaxial self-assembly of Cu nanoparticles on graphene surface: A molecular dynamics study. Comput. Mater. Sci..

[13] Muszynski R., Seger B., Kamat P.V. (2008). Decorating Graphene Sheets with Gold Nanoparticles. J. Phys. Chem. C.

[14] Zhao X.Y., Tang J.C., Yu F.X., Ye N. (2018). Preparation of graphene nanoplatelets reinforcing copper matrix composites by electrochemical deposition. J. Alloys Compd..

[15] Kim Y., Lee J., Yeom M.S., Shin J.W., Kim H., Cui Y., Kysar J.W., Hone J., Jung Y., Jeon S., Han S.M. (2013). Strengthening effect of single-atomic-layer graphene in metal-graphene nanolayered composites. Nat. Commun..

[16] Li N., Zhang L., Xu M.T., Xia T., Ruan X.W., Song S., Ma H.Z. (2016). Preparation and mechanical property of electrodeposited Al-graphene composite coating. Mater. Des..

[17] Zhao Z.Y., Li L., Bai P.K., Jin Y., Wu L.Y., Li J., Guan R.G., Qu H.Q. (2018). The Heat Treatment Influence on the Microstructure and Hardness of TC4 Titanium Alloy Manufactured via Selective Laser Melting. Materials.

[18] Zhao Z.Y., Li L., Tan L., Bai P.K., Li J., Wu L.Y., Liao H.H., Cheng Y.H. (2018). Simulation of Stress Field during the Selective Laser Melting Process of the Nickel-Based Superalloy, GH4169. Materials.

[19] Zhao Z.Y., Bai P.K., Guan R.G., Murugadoss V., Liu H., Wang X.J., Guo Z. (2018). Microstructural evolution and mechanical strengthening mechanism of Mg-3Sn-1Mn-1La alloy after heat treatments. Mater. Sci. Eng. A.

[20] Zhao Z.Y., Guan R.G., Zhang J.H., Zhao Z.Y., Bai P.K. (2017). Effects of process parameters of semisolid stirring on microstructure of Mg-3Sn-1Mn-3SiC (wt%) strip processed by rheo-rolling. Acta Metall. Sin..

[21] Cheng P., Zhao Y.H., Lu R.P., Hou H. (2018). Effect of the morphology of long-period stacking ordered phase on mechanical properties and corrosion behavior of cast Mg-Zn-Y-Ti alloy. J. Alloys Compd..

[22] Zhang Z.X., Qu S.J., Feng A.H., Shen J. (2017). Achieving grain refinement and enhanced mechanical properties in Ti-6Al-4V alloy produced by multidirectional isothermal forging. Mater. Sci. Eng. A.

[23] Zhang Z.X., Qu S.J., Feng A.H., Shen J., Chen D.L. (2017). Hot deformation behavior of Ti-6Al-4V alloy: Effect of initial microstructure. J. Alloys Compd..

[24] Zhang Z.X., Qu S.J., Feng A.H., Hu X., Shen J. (2019). Microstructural mechanisms during multidirectional isothermal forging of as-cast Ti-6Al-4V alloy with an initial lamellar microstructure. J. Alloys Compd..

[25] Li J., Zhao Z.Y., Bai P.K., Qu H.Q., Liu B., Li L., Wu L.Y., Guan R.G., Liu H., Guo Z.H. (2019). Microstructural evolution and mechanical properties of IN718 alloy fabricated by selective laser melting following different heat treatments. J. Alloys Compd..

[26] Zhao Z.Y., Misra R.D.K., Bai P.K., Gao J.F., Li Y.J., Guan R.G., Guo Z.H., Liu H. (2018). Novel process of coating Al on graphene involving organic aluminum accompanying microstructure evolution. Mater. Lett..

[27] Lisovenko A.S., Morokuma K., Timoshkin A.Y. (2015). Initial Gas Phase Reactions between Al(CH_3_)(3)/AIH(3) and Ammonia: Theoretical Study. J. Phys. Chem. A.

[28] Hou H., Wen Z.Q., Zhao Y.H., Fu L., Wang N., Han P.D. (2014). First-principles investigations on structural, elastic, thermodynamic and electronic properties of Ni_3_X (X = Al, Ga and Ge) under pressure. Intermetallics.

[29] Yang X.M., Hou H., Zhao Y.H., Yang L., Han P.D. (2014). First-principles investigation of the structural, electronic and elastic properties of Mg_x_Al_4−x_Sr (X = 0, 0.5, 1) phases. Comp. Mater. Sci..

[30] dos Santos R.B., Rivelino R., de Brito Mota F., Gueorguiev G.K., Kakanakova-Georgieva A. (2015). Dopant species with Al–Si and N–Si bonding in the MOCVD of AlN implementing trimethylaluminum, ammonia and silane. J. Phys. D Appl. Phys..

[31] Freitas R.R.Q., Gueorguiev G.K., de Brito Mota F., de Castilho C.M.C., Stafstrom S., Kakanakova-Georgieva A. (2013). Reactivity of adducts relevant to the deposition of hexagonal BN from first-principles calculations. Chem. Phys. Lett..

[32] Inagaki Y., Kozawa T. (2016). Chemical Reaction Pathways for MOVPE Growth of Aluminum Nitride. ECS J. Solid State Sci. Technol..

[33] Sangiovanni D.G., Gueorguiev G.K., Kakanakova-Georgieva A. (2018). Ab initio molecular dynamics of atomic-scale surface reactions: Insights into metal organic chemical vapor deposition of AlN on grapheme. Phys. Chem. Chem. Phys..

[34] Sadhukhan T., Samanta B., Ansari S.A., Pal S. (2016). Theoretical study of C–X [X = Cl, Br] bond activation on aluminum nanoclusters. Theor. Chem. Acc..

